# On the Helical Structure of Guanosine 5′-Monophosphate Formed at pH 5: Is It Left- or Right-Handed?

**DOI:** 10.1155/2017/6798759

**Published:** 2017-11-02

**Authors:** Gang Wu, Irene C. M. Kwan, Zhimin Yan, Yining Huang, Eric Ye

**Affiliations:** ^1^Department of Chemistry, Queen's University, 90 Bader Lane, Kingston, ON, Canada K7L 3N6; ^2^Department of Chemistry, Western University, London, ON, Canada N6A 5B7; ^3^Department of Chemistry, University of Ottawa, Ottawa, ON, Canada K1N 6N5

## Abstract

Early X-ray fiber diffraction studies have established that the spontaneous gel formation of guanosine 5′-monophosphate (5′-GMP) under slightly acidic conditions (e.g., pH 5) results from self-assembly of 5′-GMP into a helical structure in which hydrogen-bonded guanine bases form a continuous helix with 15 nucleotides per 4 turns. For more than five decades, the sense of this helix is believed to be left-handed. Using multinuclear solid-state NMR and IR spectroscopic methods, we have finally determined the long-missing structural details of this helix. First, we found that this 5′-GMP helix is right-handed containing exclusive C3′-*endo* sugar puckers. Second, we showed that the central channel of this helix is free of Na^+^ ions, which is in sharp contrast to the helix formed by 5′-GMP at pH 8 where the central channel is filled with Na^+^ ions.

## 1. Introduction

**Figure 1:**
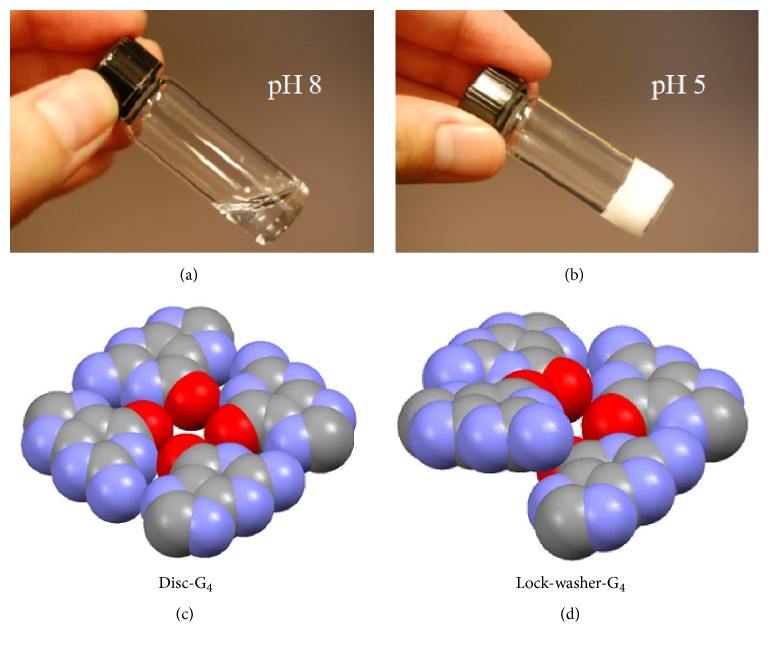
Contrasting physical appearance of 1.0 M Na_2_(5′-GMP) aqueous solution at pH 8 (a) and pH 5 (b) and the two structural motifs responsible for the 5′-GMP self-assembly: (c) planar G-quartet (disc-G_4_) and (d) open-ended G-quartet (lock-washer-G_4_).

Gel formation of guanosine 5′-monophosphate (5′-GMP) under slightly acidic conditions (e.g., pH 5) was first discovered by Bang in 1910 [1]. However, it was not until 50 years later that the structural basis of such 5′-GMP gel was examined. In 1962, Gellert et al. [2] used X-ray fiber diffraction data to show that different GMP isomers form different helical structures. For 3′-GMP gel, the helical structure is formed by successive stacking of planar hydrogen-bonded guanine tetramers now known as the G-quartets on top of each other. For 5′-GMP gel formed at pH 5, in contrast, the planar (disc-like) G-quartet is broken at one side forming a lock-washer-like structure which is further hydrogen bonded into a continuous helix; see Figure 1. In 1975, Sasisekharan et al. [3] further investigated the helical structure formed by 5′-GMP at pH 5 (i.e., 5′-GMP gel) and reported atomic coordinates for a left-handed 15/4 helix model. However, these authors also noted in the paper that* “[b ]ecause the helix is not constrained by a continuous covalently bonded backbone, both right- and left-handed helices of the 15/4 model can be constructed. Although stereochemically quite different, they are both acceptable. …For arbitrary reasons, we have selected for detailed examination a left-handed helix*….*”* Therefore, it appears that, on the basis of the original fiber X-ray diffraction data alone, there is no particular reason to favor a left-handed helix over a right-handed one. However, this arbitrary choice of the helical structure has been overlooked in the literature so that, even in classic treatises of nucleic acid structures such as that by Saenger [4], this helix is described as left-handed. It is also clear from the study of Sasisekharan et al. [3] that whether the acidic 5′-GMP helix is left- or right-handed depends critically on the sugar pucker conformation. That is, a C2′-*endo* sugar pucker would favor a left-handed helix but a C3′-*endo* conformation would result in a right-handed helix. However, because 5′-GMP gels are difficult to study with common spectroscopic techniques, the question regarding its exact helical structure has never been fully addressed.

In 2009, we used solution NMR techniques to obtain structural details of the helix formed by 5′-GMP at pH 8 [5]. As shown in Figure 1, the physical appearance of the 5′-GMP solution depends critically on the pH. At pH 8, the 5′-GMP solution appears as a normal liquid, whereas, at pH 5, it becomes a gel. The key findings of our earlier study of the 5′-GMP helix formed at pH 8 are as follows. First, the central structural motif of the helix is the disc-like G_4_. Second, the 5′-GMP molecules take alternating C2′-*endo* and C3′-*endo* sugar pucker conformation along the helical strand. Third, the helix is right-handed. Fourth, the central channel of the helix is filled with Na^+^ ions each being sandwiched between two disc-like G_4_s. In contrast, as Sasisekharan et al. [3] proposed, the central structural motif of the helix formed by 5′-GMP at pH 5 is a lock-washer-like G_4_ structure, as illustrated in Figure 1. However, other details about this helix are not known. Since 5′-GMP forms gel at pH 5, conventional solution NMR techniques are not applicable. In this work, we applied solid-state NMR and IR methods to obtain structural details about the helical structure formed by 5′-GMP at pH 5 (5′-GMP gels). In particular, we set out to address key questions concerning sugar pucker conformation, phosphate-phosphate interaction, phosphate-base interaction, and metal ion binding environment around the helical structure.

## 2. Experimental Sections

Hydrated disodium salt of guanosine 5′-monophosphate (purity > 99%) was obtained from Sigma-Aldrich (Ontario, Canada). The 5′-GMP gel sample was prepared by acidifying 1.0 M Na_2_(5′-GMP) aqueous solution to pH 5 with acetic acid. Before the solid-state NMR experiments, the gel was gently dried with a stream of N_2_. The 1D ^1^H MAS and 2D ^1^H double quantum (DQ) NMR experiments were performed at 21.1 T with a Bruker 1.3 mm HX probe with a sample spinning frequency of 62.5 kHz. The back-to-back (BABA) recoupling sequence [7] was used for the ^1^H DQ experiments with the excitation time being set to one rotor period. The recycle time employed was 8 s. The 2D ^1^H  →  ^31^P HETCOR experiments were performed at 21.1 T with a Bruker 2.4-mm MAS probe. The sample spinning frequency was 33 kHz. Contact times from 0.5 to 2.0 ms were employed. Solid-state ^13^C CP/MAS NMR experiments were performed at 14.1 and 21.1 T. All ^13^C chemical shifts were referenced to that of TMS by setting the ^13^C signal of a solid sample of tetrakis (trimethylsilyl) silane (TKS) to 3.50 ppm. Solid-state ^31^P NMR experiments were performed on a Bruker Avance-600 spectrometer operating at 242.96 MHz for ^31^P. All ^31^P chemical shifts were referenced to 85% H_3_PO_4_ (aq). Solid-state ^23^Na NMR experiments were performed on a Bruker Avance-500 spectrometer operating at 132.72 MHz for ^23^Na nuclei with the following parameters: sample spinning, 10 kHz; ^1^H decoupling, 65 kHz; recycle time, 10 s; 64 transients. All ^23^Na chemical shifts were referenced to NaCl (aq) at *δ* = 0.0 ppm by setting the ^23^Na signal of NaCl(s) to 7.21 ppm. ^23^Na{^31^P} REDOR experiments using the original version of the pulse sequence [8] were performed on a Varian/Chemagnetics Infinity-Plus 400 WB spectrometer operating at a magnetic field strength of 9.4 T. The ^31^P and ^23^Na resonance frequencies at this field strength are 161.72 and 105.67 MHz, respectively. All MAS spectra were acquired using a Varian/Chemagnetics T3 4-mm triple-tuned MAS probe. Typical RF power levels corresponded to 180° pulse lengths of 7.0 and 7.8 *μ*s for ^23^Na and ^31^P nuclei, respectively. A total of 512 transients were accumulated for each REDOR measurement. The sample spinning rate was kept constant at 10000 ± 2 Hz. The recycle delay was 0.2 s.

## 3. Results and Discussion

**Figure 2:**
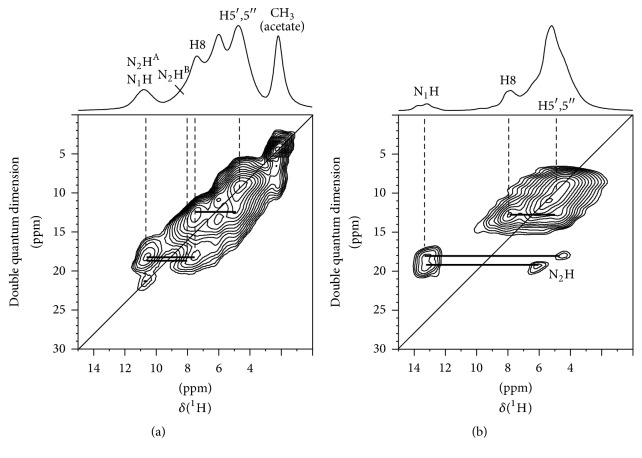
2D ^1^H DQ NMR spectra of (a) dried 5′-GMP gel formed at pH 5 and (b) Na_2_(5′-GMP)·7H_2_O (orthorhombic). The corresponding 1D ^1^H NMR spectra are shown at the top. All ^1^H NMR spectra were obtained under the MAS condition with a sample spinning frequency of 62.5 kHz at 21.1 T.

To assess the basic self-assembled structure of 5′-GMP gel formed at pH 5, we first obtained its ^1^H solid-state NMR spectra under very fast MAS conditions at an ultrahigh magnetic field, 21.1 T (900 MHz for ^1^H). For comparison, we also reported the corresponding ^1^H NMR spectra for crystalline Na_2_(5′-GMP)·7H_2_O (orthorhombic). As seen in Figure 2, for the acidic 5′-GMP gel sample, the N_1_H and N_2_H^A^ signals appear at about 10.6 ppm, suggesting that both protons are involved in strong hydrogen bonding. The DQ signals connecting N_2_H^A^ and H8 provide the most direct evidence for G_4_ formation, although this feature alone cannot reliably distinguish between the planar disk-G_4_ and lock-washer-G_4_ motifs (vide infra). Interestingly, two N_1_H signals are seen for Na_2_(5′-GMP)·7H_2_O (orthorhombic). This observation is consistent with the crystal structure of the compound where there are two distinct 5′-GMP molecules in the asymmetric unit [9]. This doubling of the signals is more evident in the ^13^C CP/MAS spectrum of Na_2_(5′-GMP)·7H_2_O (orthorhombic) (see Figure S1 in the Supporting Information, available online at https://doi.org/10.1155/2017/6798759). Furthermore, for Na_2_(5′-GMP)·7H_2_O (orthorhombic), the N_1_H signals appear at about 13.5 ppm, whereas the corresponding N_2_H signals are between 4 and 6 ppm. These observed ^1^H chemical shifts are in agreement with the crystal structure of Na_2_(5′-GMP)·7H_2_O (orthorhombic) which shows that N_1_H forms a strong hydrogen bond with ^−^O-P (the two N⋯O distances are 2.76 and 2.79 Å) and the N_2_H groups are only weakly hydrogen bonded to water molecules (two N⋯O_W_ distances are 2.91 and 2.95 Å) [9]. It is interesting to note that both acidic 5′-GMP gel and Na_2_(5′-GMP)·7H_2_O exhibit DQ signals between H8 and H5′,5′′, consistent with the guanine base being in the* anti*-conformation. Now, while the ^1^H solid-state NMR data confirm G4 formation for the acidic 5′-GMP gel, they provide no information about the sense of the helix.

As mentioned earlier, on the basis of modeling performed by Sasisekharan et al. [3], whether the acidic 5′-GMP helix is left- or right-handed depends critically on the sugar pucker conformation. To answer this question, we utilized a well-established approach in using ^13^C chemical shifts of the sugar carbons as a means of determining the sugar pucker conformation. In particular, Harbison and coworkers [10, 11] showed that, for RNA nucleosides and nucleotides, one can combine the ^13^C chemical shifts observed for the ribose moiety into the following two canonical coordinates:

(1) can1=0.179δC1′−0.225δC4′−0.0585δC5′,can2=−0.0605δC2′+δC3′−0.0556δC4′−0.0524δC5′.

Then any data point appearing in the can1-can2 plot can be used to determine the sugar pucker conformation (can1 > −6.77 for C3′-*endo* and can1 < −6.77 for C2′-*endo*) as well as the exocyclic *γ*-torsion angle (can2 < −16.82 for* gt* and can2 > −16.82 for* gg*). Later, Ohlenschläger et al. [12] applied this approach to analyze a total of 429 known RNA structures and showed that the reliability of this approach for purine nucleotides is 93-94% (see Figure S2 in the Supporting Information).

**Figure 3:**
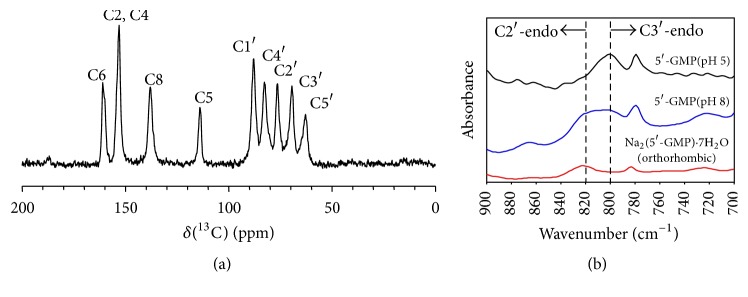
(a) ^13^C CP/MAS NMR spectrum of dried 5′-GMP gel formed at pH 5. (b) The signature region of IR spectra revealing sugar pucker conformation for 5′-GMP gel (pH 5), 5′-GMP (pH 8), and Na_2_(5′-GMP)·7H_2_O (orthorhombic).

Figure 3(a) shows the solid-state ^13^C CP/MAS NMR spectrum of acidic 5′-GMP gel where the observed ^13^C chemical shifts for sugar carbons C1′, C2′, C3′, C4′, and C5′ are 87.9, 76.3, 69.3, 82.2, and 62.8 ppm, respectively. This assignment was further confirmed by DFT calculations on the ^13^C chemical shifts for a model 5′-GMP molecule. These values yield can1 = −6.43 and can2 = −16.67 for the acidic 5′-GMP gel. Now the fact that can1 > −6.77 and can2 > −16.82 for the acidic 5′-GMP gel strongly suggests that the sugar pucker conformation is exclusively C3′-*endo* with the exocyclic *γ*-torsion angle being in the* gg* conformation [10–12]; see Figure S2 in the Supporting Information. These canonical coordinates are quite different from those for Na_2_(5′-GMP)·7H_2_O (orthorhombic) and 5′-GMP self-assembly formed at pH 8; also see Figure S2 in the Supporting Information. This new information about the C3′-*endo* sugar pucker conformation means that the continuous helix of the acidic 5′-GMP gel is right-handed. To further confirm the C3′-*endo* sugar pucker conformation determined above, we recorded FTIR spectra for three 5′-GMP samples. Some time ago, Tajmir-Riahi [13] showed that the P-O-5′-ribose stretch frequency can be used as the signature for the sugar pucker conformation for guanylic acid and its salts: 800 cm^–1^ for C3′-*endo* and 820 cm^–1^ for C2′-*endo*. As seen from Figure 3(b), the acidic 5′-GMP gel sample indeed displays a peak at 800 cm^–1^, confirming the aforementioned C3′-*endo* sugar pucker conformation. In comparison, the FTIR spectrum of the 5′-GMP self-assembly formed at pH 8 exhibits both 800 and 820 cm^–1^ peaks of equal intensity. This is in agreement with the earlier observation that the helical structure of 5′-GMP formed at pH 8 consists of alternating C3′-*endo* and C2′-*endo* sugar pucker conformation [5]. For Na_2_(5′-GMP)·7H_2_O (orthorhombic), the observation of a peak at 820 cm^–1^ is in agreement with its crystal structure where the ribose is in the C2′-*endo* conformation [9]. Therefore, the FTIR data shown in Figure 3(b) are fully consistent with the results on sugar pucker conformation obtained from the ^13^C chemical shift analysis. Now, combining the C3′-*endo* sugar pucker conformation with the helical parameters reported by Sasisekharan et al. [3], we can readily build a right-handed 15/4 helix model; see Figure S3 and Table S1 in the Supporting Information for atomic coordinates.

**Figure 4:**
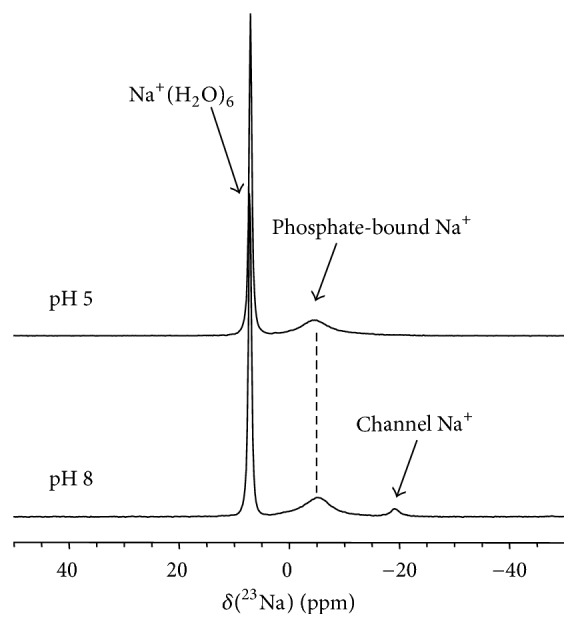
^23^Na MAS NMR spectra of the 5′-GMP samples prepared at pH 5 and pH 8.

**Figure 5:**
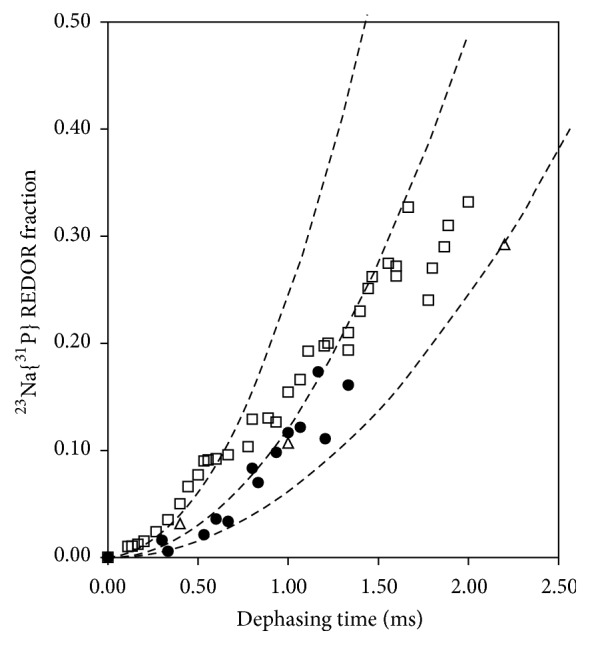
^23^Na{^31^P} REDOR results obtained for (□) 5′-GMP (pH 5), (●) 5′-GMP (pH 8), and (∆) double-stranded calf thymus DNA (A-form). The dash lines are calculated using Δ*S*/*S* = (4/3*π*^2^)(*NT*_*r*_)^2^ *M*_2_, where *NT*_*r*_ is the dephasing time and *M*_2_ is the second moment of the ^23^Na-^31^P dipolar interactions [6]. The three *M*_2_ values used in the calculations are 1.80, 0.90, and 0.45 × 10^6^ s^–2^, corresponding to a Na-P distance between 3.1, 3.4, and 3.7 Å, respectively.

**Figure 6:**
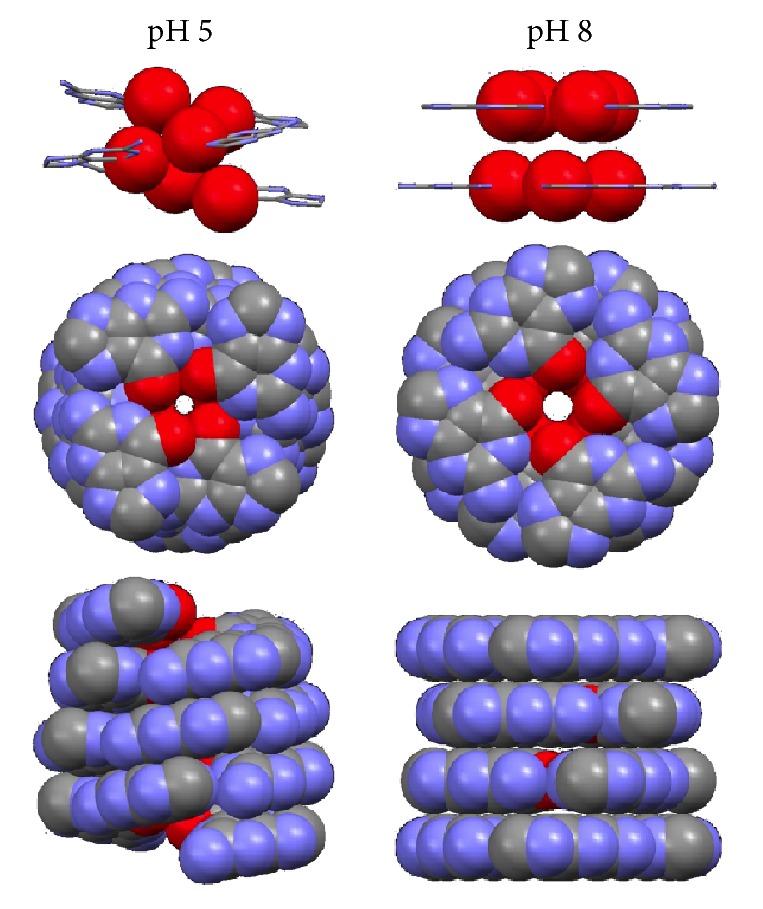
Different arrangements of the guanine bases in the helical structures of 5′-GMP self-assembly formed under acidic (pH 5) and neutral (pH 8) conditions. Both helices are right-handed.

Since metal ion binding is an integral part of G-quadruplex formation [14–18], we further investigated how Na^+^ ions are bound to the acidic 5′-GMP helical structure.  Figure 4 shows the solid-state ^23^Na NMR spectra obtained for the acidic 5′-GMP gel as well as for a neutral 5′-GMP self-assembly sample for comparison. The two ^23^Na NMR signals observed for the acidic 5′-GMP gel can be readily assigned: the sharp signal at 7.2 ppm is due to fully hydrated Na^+^ ions and the signal centered at −5.0 ppm is from phosphate-bound Na^+^ ions. To further confirm the phosphate-bound nature of the signal at −5.0 ppm, we performed ^23^Na{^31^P} rotational-echo double resonance (REDOR) [8] experiments. As shown in Figure 5, the ^23^Na{^31^P} REDOR results obtained for the acidic 5′-GMP gel are quite similar to those for neutral 5′-GMP and for double-stranded calf thymus DNA in the dry state (A-form) [19]. Thus the ^23^Na{^31^P} REDOR results confirmed unambiguously that the ^23^Na NMR signal at −5 ppm arises from Na^+^ ions bound to the phosphate group. The most striking feature in the ^23^Na NMR spectrum of acidic 5′-GMP gel is the absence of any signal at ca. −18 ppm, which is the established spectral signature for Na^+^ ions residing inside a G-quadruplex channel [20–22]. This observation immediately suggests that there is no Na^+^ ion inside the central channel of the continuous helix formed by 5′-GMP at pH 5! This aspect of the helix, though totally unexpected, can be readily understood on the basis of our structural model. As seen from Figure 6, when a disc-like G_4_ is twisted into a lock-washer-like G_4_, the size of the central cavity surrounded by carbonyl oxygen atoms is significantly reduced. As a result, Na^+^ ions can no longer fit into this cavity. The diameter of the central channel is reduced by nearly 50% for the acidic 5′-GMP helix compared with that of the neutral 5′-GMP helix, as clearly seen from the top view of the channel shown in Figure 6. This observation is consistent with the fact that 5′-GMP gel formation at pH 5 is not sensitive to the nature of monovalent cations (Na^+^, K^+^, or NH_4_^+^) present in solution. It is also worth noting that the helical structure of acidic 5′-GMP gel is remarkably similar to that found for 8-oxoguanosine reported recently by Giorgi et al. [23], despite the very different hydrogen bonding schemes in these two systems. Here we further comment on the role that the central cations play in G-quadruplex systems consisting planar disc-like G-quartets. While it is commonly accepted that the central cation is to reduce the repulsion between carbonyl oxygen atoms from G-quartets, it is important to point out that it is primarily the repulsions between carbonyl oxygen atoms from adjacent planar G-quartets, not from within the same G-quartet, which requires further stabilization from a cation. The main evidence for this view is the fact that whereas a cation-free or “empty” G-quartet was observed [24], an “empty” G-octamer has never been reported. Now when the helix is made of lock-washer-like G_4_, there is no longer any repulsion between carbonyl oxygen atoms along the helical axis, thus making it unnecessary to have a cation inside the central channel.

Now let us turn attention to the phosphate group in the acidic 5′-GMP helix. Since the phosphate group of 5′-GMP has a p*K*_a2_ of 7.5, it is doubly charged at pH 8 but only singly charged at pH 5. We discovered in an earlier study [5] that two types of phosphate groups are present in the 5′-GMP helix formed at pH 8 and they are possibly bridged by a Na^+^ ion. For the 5′-GMP helix formed at pH 5, our model suggests that singly charged phosphate groups form a continuous hydrogen-bonded chain along the helical “strand” (i.e., ⋯HO-P_*i*_-O^−^⋯HO-P_*i*+1_-O^−^⋯). This type of hydrogen bond chains are commonly observed in the crystal structures of ammonium hydrogen alkylphosphates [25]. Because of this strong hydrogen bonding interaction, the P⋯P distance is significantly shorter in the acidic 5′-GMP helix, 5.2 Å, than in the neutral 5′-GMP helix (6.7 and 7.2 Å) [5]. The solid-state ^31^P NMR spectrum of acidic 5′-GMP gel exhibits a sharp peak at 1.3 ppm (vide infra), suggesting that all phosphate groups are equivalent. This is in contrast to the situation seen in the neutral 5′-GMP helix where two different phosphate groups are present with the ^31^P chemical shifts being 3.7 and 5.2 ppm [5]. Another important structural feature in the acidic 5′-GMP helix is the possible formation of a phosphate-base hydrogen bond, as first noted by Sasisekharan et al. [3]. In particular, the *i*th phosphate group can be hydrogen-bonded to the exocyclic amino group of the (*i* + 3)th guanine base (i.e., N_2_-H^B^⋯O=P) along the helical strand. In our model, the N_2_⋯O(P) distance is ca. 2.82 Å.

**Figure 7:**
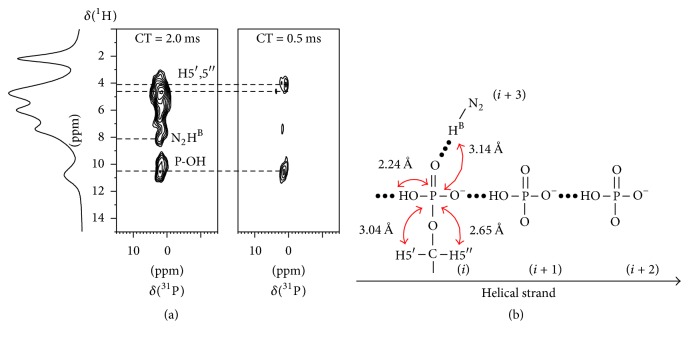
(a) 2D ^1^H  →  ^31^P HETCOR NMR spectra of the acidic 5′-GMP gel sample obtained at two different contact times (CT). (b) Predicted short contacts between the phosphate atom and several protons in the acidic 5′-GMP helical model.

To search for further spectroscopic evidence for the aforementioned two types of hydrogen bonding interactions involving the phosphate group (i.e., ⋯HO-P_*i*_-O^−^⋯HO-P_*i*+1_-O^−^⋯ and N_2_-H^B^⋯O=P), we performed 2D ^1^H  →  ^31^P HETCOR experiments. As seen in Figure 7(a), at a short contact time of 0.5 ms, two cross peaks were observed. The weaker cross peak with *δ*(^1^H) of 4.1 ppm clearly arises from the short contacts between the phosphorus atom and H5′,5′′ (2.65 and 3.04 Å), as illustrated in Figure 7(b). The stronger ^1^H-^31^P cross peak with *δ*(^1^H) of 10.5 ppm is an interesting discovery, because we have already attributed, in the earlier discussion, N_1_H and N_2_H^A^ to this overlapping signal. Now we see that a third signal, which displays the shortest contact with the P atom, also appears in this ^1^H chemical shift region. This new signal must be due to the P-OH group (the H-P distance is ca. 2.24 Å in our model); see Figure 7(b). As seen in Figure 7(a), a new cross peak corresponding to the N_2_H^B^ group emerges at a longer contact time (2 ms). This is consistent with our model where the P atom is predicted to be 3.14 Å away from N_2_H^B^, due to the formation of a N_2_-H^B^⋯O=P hydrogen bond. This hydrogen bond further explains why the ^1^H chemical shift of N_2_-H^B^, ca. 8 ppm, is considerably higher than those seen in the neutral 5′-GMP helix, 5.12 and 4.29 ppm [5]. Close inspection of the acidic 5′-GMP helix suggests that the formation of this strong N_2_-H^B^⋯·O=P hydrogen bond causes a tilting of the guanine base, thus making it more difficult to form a planar disc-like G_4_. We postulate that the hydrogen bonding interactions between singly charged phosphate groups (⋯HO-P_*i*_-O^−^⋯HO-P_*i*+1_-O^−^⋯) and between phosphate and guanine (N_2_-H^B^⋯O=P) are the driving forces for the self-assembly of 5′-GMP into a continuous helix at pH 5.

## 4. Conclusion

In this work, we have obtained new structural details about the helical structure formed by 5′-GMP at pH 5. Contrary to the common assumption, we showed that this helix is composed of 5′-GMP molecules exclusively in C3′-*endo* sugar pucker conformation and consequently is right-handed. In addition, we found that the central channel of the helix is free of Na^+^ ions. In many aspects, this helix is drastically different from the one formed by 5′-GMP at pH 8. Remarkably, two different helices can form by the same molecule at just slightly different pH values. Of course, at pH 5 and 8, the charge state of the phosphate group would be different. The present study has provided another example where mononucleotides can self-associate into a helix* in the absence of phosphodiester bonds*. The solid-state NMR strategies demonstrated in this study can be applied to similar gels formed by other nucleosides and nucleotides.

## Supplementary Material

Figure S1 shows the 13C CP/MAS NMR spectrum of orthorhombic Na2(5'-GMP).7H2O.
Figure S2 shows the can1-can2 plot for RNA.
Figure S3 displays side- and top-views of the right-handed 15/4 helix formed by 5'-GMP at pH5.
Table S1 lists atomic coodinates for the right-handed 15/4 helix formed by 5'-GMP at pH5.
