# Supplementary material for: On the Helical Structure of Guanosine 5′-Monophosphate Formed at pH 5: Is It Left- or Right-Handed?
Source: J Nucleic Acids. 2017 Nov 2;2017:6798759. doi: 10.1155/2017/6798759 (PMC5688352; doi:10.1155/2017/6798759)
Supplement: Supplementary file 1 — Figure S1 shows the 13C CP/MAS NMR spectrum of orthorhombic Na2(5'-GMP).7H2O. Figure S2 shows the can1-can2 plot for RNA. Figure S3 displays side- and top-views of the right-handed 15/4 helix formed by 5'-GMP at pH5. Table S1 lists atomic coodinates for the right-handed 15/4 helix formed by 5'-GMP at pH5. [file 6798759.f1.pdf]

On the Helical Structure of Guanosine 5'-Monophosphate Formed at pH 5: Is It Left- or Right-Handed?

Gang Wu,*[1] Irene C. M. Kwan,[1] Zhimin Yan,[2] Yining Huang,[2] and Eric Ye[3]

[1]Department of Chemistry, Queen's University, Kingston, Ontario, K7L 3N6, Canada;
[2]Department of Chemistry, The University of Western Ontario, London, Ontario, N6A 5B7, Canada;
[3]Department of Chemistry, University of Ottawa, Ottawa, Ontario, K1N 6N5, Canada
(Email: wugang@queensu.ca)

Supporting Information

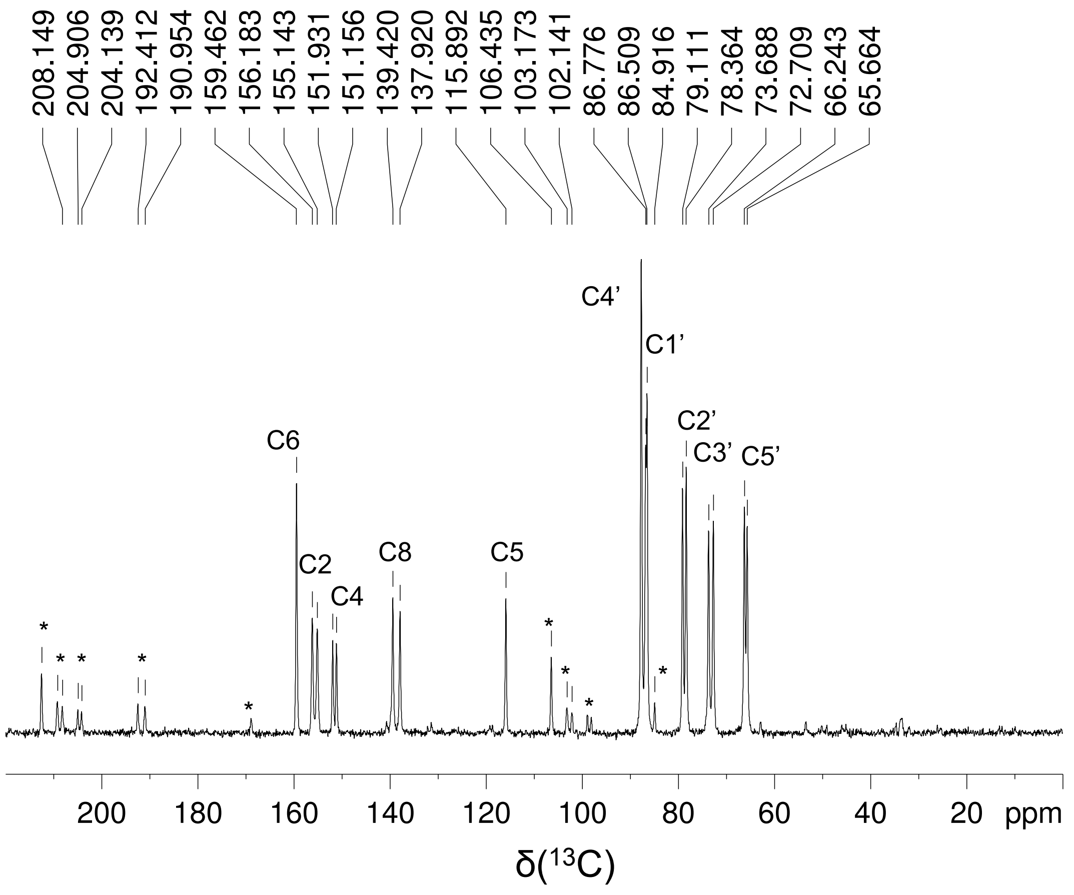

Figure S1. Solid-state ^{13}C CP/MAS NMR spectrum of Na_2(5'-GMP)·7H_2O (orthorhombic) showing the doubling of each signal due to the presence of two distinct 5'-GMP molecules in the asymmetric unit of the crystal lattice. The spectrum was obtained at 21.1 T with a 4-mm Bruker HCN probe. The sample spinning frequency was 12 kHz. A recycle time of 20 s was used. All spinning sidebands are marked by *.

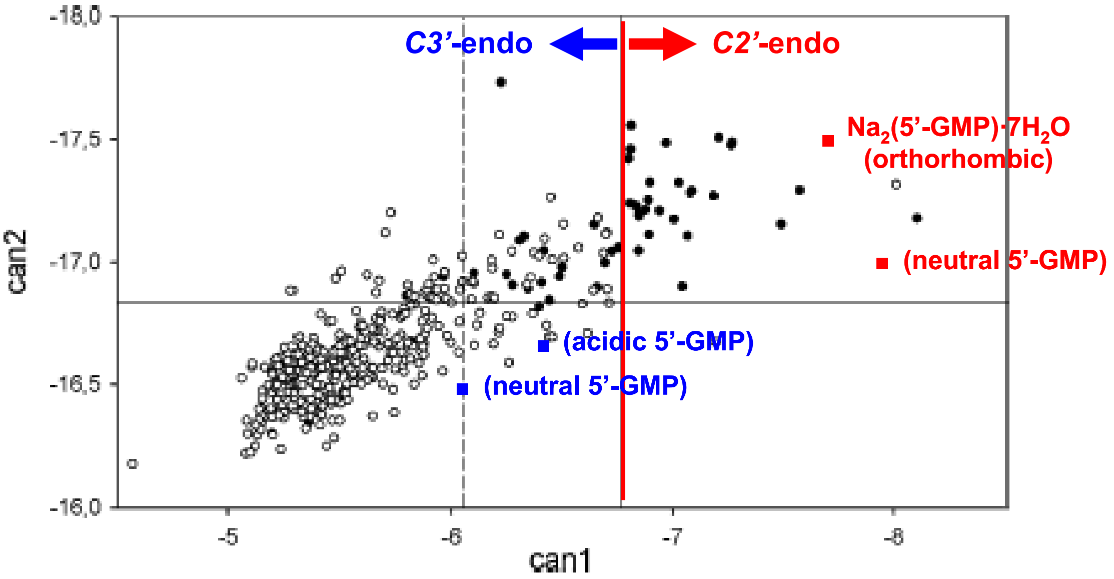

$$\mathrm{can1} = 0.179\delta_{C1'} - 0.225\delta_{C4'} - 0.0585\delta_{C5'}$$

$$\mathrm{can2} = -0.0605\delta_{(C2'+C3')} - 0.0556\delta_{C4'} - 0.0524\delta_{C5'}$$

Figure S2. Illustration of the approach used to determine sugar pucker conformation from ^{13}C chemical shifts. The background plot was reproduced from [11] [O. Ohlenschläger, S. Haumann, R. Ramachandran, M. Görlach, *J. Biomol. NMR* vol. 42, pp. 139-142, 2008].

(a) (b)

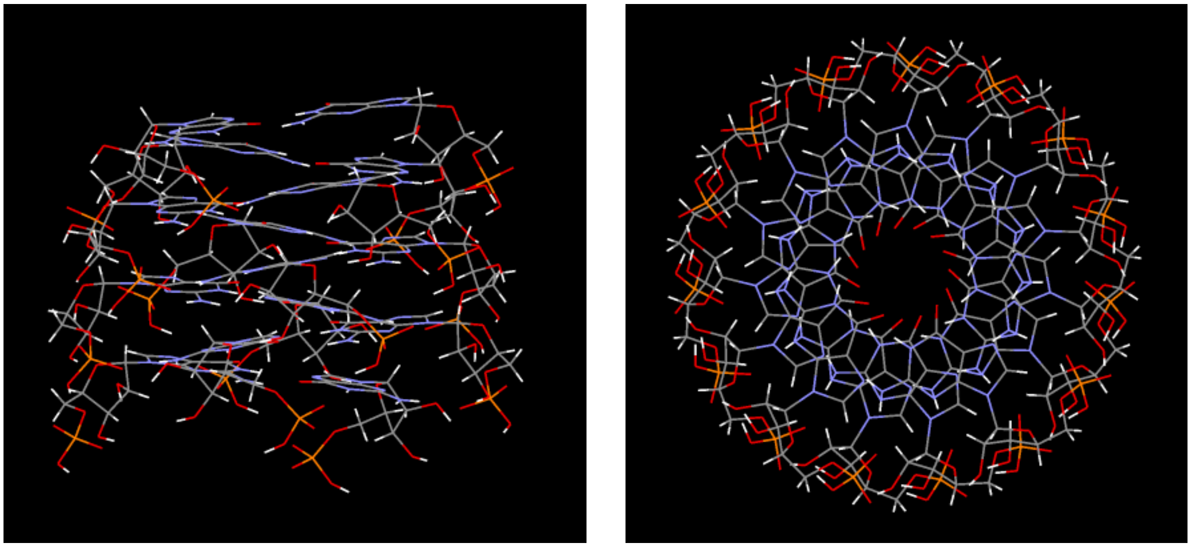

Figure S3. (a) Side- and (b) top-views of the right-handed 15/4 helix formed by 5′-GMP at pH 5.

Table S1. Atomic coordinates of the right-handed 15/4 helix formed by 5’-GMP at pH 5.

HETATM	1	N	1	1.665	2.907	-4.000	N
HETATM	2	H	1	0.788	2.775	-3.848	H
HETATM	3	C	1	2.140	4.218	-3.900	C
HETATM	4	N	1	3.500	4.512	-3.940	N
HETATM	5	C	1	4.197	3.398	-4.200	C
HETATM	6	C	1	3.893	2.114	-4.340	C
HETATM	7	C	1	2.421	1.805	-4.310	C
HETATM	8	N	1	4.949	1.234	-4.550	N
HETATM	9	C	1	5.963	2.065	-4.600	C
HETATM	10	H	1	6.854	1.780	-4.815	H
HETATM	11	N	1	5.639	3.429	-4.310	N
HETATM	12	O	1	1.930	0.661	-4.340	O
HETATM	13	N	1	1.274	5.186	-3.520	N
HETATM	14	H	1	0.417	4.977	-3.339	H
HETATM	15	H	1	1.554	6.038	-3.448	H
HETATM	16	C	1	6.636	4.606	-4.296	C
HETATM	17	H	1	6.553	5.156	-5.077	H
HETATM	18	C	1	6.401	5.455	-3.047	C
HETATM	19	H	1	5.495	5.399	-2.736	H
HETATM	20	C	1	7.387	4.846	-2.057	C
HETATM	21	H	1	7.023	4.032	-1.701	H
HETATM	22	C	1	8.566	4.527	-2.944	C
HETATM	23	H	1	9.086	5.323	-3.083	H
HETATM	24	C	1	9.481	3.399	-2.449	C
HETATM	25	H	1	9.973	3.696	-1.681	H
HETATM	26	H	1	10.109	3.169	-3.137	H
HETATM	27	O	1	7.979	4.109	-4.204	O
HETATM	28	O	1	6.774	6.785	-3.371	O
HETATM	29	H	1	6.523	7.422	-2.575	H
HETATM	30	O	1	7.737	5.726	-0.989	O
HETATM	31	H	1	6.950	6.383	-0.811	H
HETATM	32	O	1	8.694	2.248	-2.108	O
HETATM	33	O	1	10.319	0.371	-1.505	O
HETATM	34	O	1	9.930	2.107	0.209	O
HETATM	35	O	1	8.123	0.351	-0.483	O
HETATM	36	P	1	9.240	1.261	-0.936	P
HETATM	37	H	1	9.348	2.892	0.593	H
HETATM	38	N	1	2.717	-1.960	-4.883	N
HETATM	39	H	1	2.677	-1.074	-4.731	H
HETATM	40	C	1	3.971	-2.569	-4.783	C
HETATM	41	N	1	4.121	-3.952	-4.823	N
HETATM	42	C	1	2.941	-4.529	-5.083	C
HETATM	43	C	1	1.695	-4.093	-5.223	C
HETATM	44	C	1	1.542	-2.596	-5.193	C
HETATM	45	N	1	0.710	-5.051	-5.433	N
HETATM	46	C	1	1.430	-6.146	-5.483	C
HETATM	47	H	1	1.054	-7.003	-5.698	H
HETATM	48	N	1	2.821	-5.967	-5.193	N
HETATM	49	O	1	0.456	-1.989	-5.223	O
HETATM	50	N	1	5.024	-1.809	-4.403	N
HETATM	51	H	1	4.906	-0.935	-4.222	H
HETATM	52	H	1	5.842	-2.177	-4.331	H
HETATM	53	C	1	3.887	-7.081	-5.179	C

HETATM	54	H	1	4.442	-7.056	-5.960		H
HETATM	55	C	1	4.756	-6.936	-3.930		C
HETATM	56	H	1	4.795	-6.029	-3.619		H
HETATM	57	C	1	4.047	-7.853	-2.940		C
HETATM	58	H	1	3.276	-7.406	-2.584		H
HETATM	59	C	1	3.607	-8.993	-3.827		C
HETATM	60	H	1	4.344	-9.592	-3.966		H
HETATM	61	C	1	2.389	-9.784	-3.332		C
HETATM	62	H	1	2.633	-10.305	-2.564		H
HETATM	63	H	1	2.095	-10.385	-4.020		H
HETATM	64	O	1	3.252	-8.365	-5.087		O
HETATM	65	O	1	6.040	-7.446	-4.254		O
HETATM	66	H	1	6.700	-7.264	-3.458		H
HETATM	67	O	1	4.886	-8.293	-1.872		O
HETATM	68	H	1	5.622	-7.580	-1.694		H
HETATM	69	O	1	1.327	-8.881	-2.991		O
HETATM	70	O	1	-0.709	-10.301	-2.388		O
HETATM	71	O	1	1.057	-10.096	-0.674		O
HETATM	72	O	1	-0.500	-8.115	-1.366		O
HETATM	73	P	1	0.288	-9.322	-1.819		P
HETATM	74	H	1	1.899	-9.599	-0.290		H
HETATM	75	N	1	-2.233	-2.497	-5.766		N
HETATM	76	H	1	-1.348	-2.551	-5.614		H
HETATM	77	C	1	-2.970	-3.681	-5.666		C
HETATM	78	N	1	-4.362	-3.686	-5.706		N
HETATM	79	C	1	-4.812	-2.451	-5.966		C
HETATM	80	C	1	-4.247	-1.258	-6.106		C
HETATM	81	C	1	-2.743	-1.262	-6.076		C
HETATM	82	N	1	-5.097	-0.178	-6.316		N
HETATM	83	C	1	-6.262	-0.780	-6.366		C
HETATM	84	H	1	-7.074	-0.316	-6.581		H
HETATM	85	N	1	-6.229	-2.182	-6.076		N
HETATM	86	O	1	-2.025	-0.245	-6.106		O
HETATM	87	N	1	-2.324	-4.808	-5.286		N
HETATM	88	H	1	-1.443	-4.782	-5.105		H
HETATM	89	H	1	-2.775	-5.583	-5.214		H
HETATM	90	C	1	-7.448	-3.126	-6.062		C
HETATM	91	H	1	-7.482	-3.681	-6.843		H
HETATM	92	C	1	-7.395	-4.005	-4.813		C
HETATM	93	H	1	-6.497	-4.139	-4.502		H
HETATM	94	C	1	-8.233	-3.204	-3.823		C
HETATM	95	H	1	-7.708	-2.484	-3.467		H
HETATM	96	C	1	-9.320	-2.647	-4.710		C
HETATM	97	H	1	-9.994	-3.317	-4.849		H
HETATM	98	C	1	-9.980	-1.353	-4.215		C
HETATM	99	H	1	-10.524	-1.541	-3.447		H
HETATM	100	H	1	-10.547	-0.998	-4.903		H
HETATM	101	O	1	-8.659	-2.360	-5.970		O
HETATM	102	O	1	-8.036	-5.228	-5.137		O
HETATM	103	H	1	-7.924	-5.904	-4.341		H
HETATM	104	O	1	-8.758	-3.992	-2.755		O
HETATM	105	H	1	-8.126	-4.799	-2.577		H
HETATM	106	O	1	-8.971	-0.391	-3.874		O
HETATM	107	O	1	-10.171	1.782	-3.271		O
HETATM	108	O	1	-10.151	0.004	-1.557		O
HETATM	109	O	1	-8.018	1.346	-2.249		O
HETATM	110	P	1	-9.301	0.688	-2.702		P

HETATM	111	H	1	-9.745	-0.885	-1.173	H
HETATM	112	N	1	-2.250	2.482	-6.649	N
HETATM	113	H	1	-2.396	1.607	-6.497	H
HETATM	114	C	1	-3.350	3.339	-6.549	C
HETATM	115	N	1	-3.210	4.723	-6.589	N
HETATM	116	C	1	-1.935	5.042	-6.849	C
HETATM	117	C	1	-0.808	4.356	-6.989	C
HETATM	118	C	1	-0.969	2.860	-6.959	C
HETATM	119	N	1	0.356	5.088	-7.199	N
HETATM	120	C	1	-0.121	6.309	-7.249	C
HETATM	121	H	1	0.425	7.069	-7.464	H
HETATM	122	N	1	-1.519	6.423	-6.959	N
HETATM	123	O	1	-0.032	2.040	-6.989	O
HETATM	124	N	1	-4.538	2.814	-6.169	N
HETATM	125	H	1	-4.605	1.935	-5.988	H
HETATM	126	H	1	-5.262	3.344	-6.097	H
HETATM	127	C	1	-2.330	7.734	-6.945	C
HETATM	128	H	1	-2.878	7.825	-7.726	H
HETATM	129	C	1	-3.210	7.773	-5.696	C
HETATM	130	H	1	-3.437	6.894	-5.385	H
HETATM	131	C	1	-2.326	8.523	-4.706	C
HETATM	132	H	1	-1.665	7.925	-4.350	H
HETATM	133	C	1	-1.659	9.546	-5.593	C
HETATM	134	H	1	-2.255	10.286	-5.732	H
HETATM	135	C	1	-0.302	10.067	-5.098	C
HETATM	136	H	1	-0.433	10.627	-4.330	H
HETATM	137	H	1	0.110	10.594	-5.786	H
HETATM	138	O	1	-1.442	8.858	-6.853	O
HETATM	139	O	1	-4.360	8.539	-6.020	O
HETATM	140	H	1	-5.043	8.498	-5.224	H
HETATM	141	O	1	-3.055	9.127	-3.638	O
HETATM	142	H	1	-3.923	8.583	-3.460	H
HETATM	143	O	1	0.549	8.963	-4.757	O
HETATM	144	O	1	2.836	9.929	-4.154	O
HETATM	145	O	1	1.065	10.095	-2.440	O
HETATM	146	O	1	2.176	7.833	-3.132	O
HETATM	147	P	1	1.656	9.178	-3.585	P
HETATM	148	H	1	0.138	9.784	-2.056	H
HETATM	149	N	1	2.703	1.978	-7.532	N
HETATM	150	H	1	1.849	2.215	-7.380	H
HETATM	151	C	1	3.671	2.983	-7.432	C
HETATM	152	N	1	5.033	2.698	-7.472	N
HETATM	153	C	1	5.216	1.397	-7.732	C
HETATM	154	C	1	4.416	0.348	-7.872	C
HETATM	155	C	1	2.946	0.664	-7.842	C
HETATM	156	N	1	5.023	-0.886	-8.082	N
HETATM	157	C	1	6.287	-0.539	-8.132	C
HETATM	158	H	1	6.985	-1.162	-8.347	H
HETATM	159	N	1	6.546	0.839	-7.842	N
HETATM	160	O	1	2.032	-0.181	-7.872	O
HETATM	161	N	1	3.273	4.219	-7.052	N
HETATM	162	H	1	2.405	4.377	-6.871	H
HETATM	163	H	1	3.876	4.884	-6.980	H
HETATM	164	C	1	7.935	1.509	-7.828	C
HETATM	165	H	1	8.083	2.045	-8.609	H
HETATM	166	C	1	8.066	2.380	-6.579	C
HETATM	167	H	1	7.216	2.697	-6.268	H

HETATM	168	C	1	8.719	1.422	-5.589	C
HETATM	169	H	1	8.056	0.827	-5.233	H
HETATM	170	C	1	9.667	0.652	-6.476	C
HETATM	171	H	1	10.465	1.167	-6.615	H
HETATM	172	C	1	10.044	-0.751	-5.981	C
HETATM	173	H	1	10.614	-0.680	-5.213	H
HETATM	174	H	1	10.524	-1.217	-6.669	H
HETATM	175	O	1	8.961	0.508	-7.736	O
HETATM	176	O	1	8.948	3.443	-6.903	O
HETATM	177	H	1	8.978	4.127	-6.107	H
HETATM	178	O	1	9.397	2.084	-4.521	O
HETATM	179	H	1	8.946	3.005	-4.343	H
HETATM	180	O	1	8.856	-1.483	-5.640	O
HETATM	181	O	1	9.578	-3.858	-5.037	O
HETATM	182	O	1	9.928	-2.114	-3.323	O
HETATM	183	O	1	7.563	-2.983	-4.015	O
HETATM	184	P	1	8.954	-2.607	-4.468	P
HETATM	185	H	1	9.716	-1.160	-2.939	H
HETATM	186	N	1	1.685	-2.895	-8.415	N
HETATM	187	H	1	2.009	-2.070	-8.263	H
HETATM	188	C	1	2.583	-3.962	-8.315	C
HETATM	189	N	1	2.158	-5.287	-8.355	N
HETATM	190	C	1	0.844	-5.334	-8.615	C
HETATM	191	C	1	-0.116	-4.428	-8.755	C
HETATM	192	C	1	0.353	-2.999	-8.725	C
HETATM	193	N	1	-1.406	-4.903	-8.965	N
HETATM	194	C	1	-1.193	-6.197	-9.015	C
HETATM	195	H	1	-1.885	-6.826	-9.230	H
HETATM	196	N	1	0.150	-6.598	-8.725	N
HETATM	197	O	1	-0.393	-2.002	-8.755	O
HETATM	198	N	1	3.854	-3.696	-7.935	N
HETATM	199	H	1	4.102	-2.850	-7.754	H
HETATM	200	H	1	4.452	-4.365	-7.863	H
HETATM	201	C	1	0.671	-8.050	-8.711	C
HETATM	202	H	1	1.188	-8.253	-9.492	H
HETATM	203	C	1	1.524	-8.271	-7.462	C
HETATM	204	H	1	1.928	-7.458	-7.151	H
HETATM	205	C	1	0.503	-8.820	-6.472	C
HETATM	206	H	1	-0.019	-8.098	-6.116	H
HETATM	207	C	1	-0.362	-9.682	-7.359	C
HETATM	208	H	1	0.067	-10.530	-7.498	H
HETATM	209	C	1	-1.797	-9.910	-6.864	C
HETATM	210	H	1	-1.786	-10.485	-6.096	H
HETATM	211	H	1	-2.310	-10.339	-7.552	H
HETATM	212	O	1	-0.431	-8.965	-8.619	O
HETATM	213	O	1	2.489	-9.259	-7.786	O
HETATM	214	H	1	3.166	-9.361	-6.990	H
HETATM	215	O	1	1.091	-9.563	-5.404	O
HETATM	216	H	1	2.053	-9.211	-5.226	H
HETATM	217	O	1	-2.400	-8.653	-6.523	O
HETATM	218	O	1	-4.838	-9.122	-5.920	O
HETATM	219	O	1	-3.140	-9.653	-4.206	O
HETATM	220	O	1	-3.757	-7.210	-4.898	O
HETATM	221	P	1	-3.528	-8.633	-5.351	P
HETATM	222	H	1	-2.170	-9.541	-3.822	H
HETATM	223	N	1	-3.056	-1.373	-9.298	N
HETATM	224	H	1	-2.269	-1.782	-9.146	H

HETATM	225	C	1	-4.211	-2.155	-9.198	C
HETATM	226	N	1	-5.484	-1.593	-9.238	N
HETATM	227	C	1	-5.393	-0.282	-9.498	C
HETATM	228	C	1	-4.392	0.578	-9.638	C
HETATM	229	C	1	-3.020	-0.037	-9.608	C
HETATM	230	N	1	-4.729	1.911	-9.848	N
HETATM	231	C	1	-6.038	1.834	-9.898	C
HETATM	232	H	1	-6.591	2.589	-10.113	H
HETATM	233	N	1	-6.578	0.540	-9.608	N
HETATM	234	O	1	-1.950	0.600	-9.638	O
HETATM	235	N	1	-4.079	-3.447	-8.818	N
HETATM	236	H	1	-3.263	-3.781	-8.637	H
HETATM	237	H	1	-4.806	-3.971	-8.746	H
HETATM	238	C	1	-8.076	0.174	-9.594	C
HETATM	239	H	1	-8.332	-0.319	-10.375	H
HETATM	240	C	1	-8.385	-0.651	-8.345	C
HETATM	241	H	1	-7.619	-1.138	-8.034	H
HETATM	242	C	1	-8.824	0.421	-7.355	C
HETATM	243	H	1	-8.052	0.866	-6.999	H
HETATM	244	C	1	-9.591	1.373	-8.242	C
HETATM	245	H	1	-10.479	1.034	-8.381	H
HETATM	246	C	1	-9.668	2.823	-7.747	C
HETATM	247	H	1	-10.241	2.872	-6.979	H
HETATM	248	H	1	-10.041	3.379	-8.435	H
HETATM	249	O	1	-8.870	1.366	-9.502	O
HETATM	250	O	1	-9.468	-1.508	-8.669	O
HETATM	251	H	1	-9.640	-2.170	-7.873	H
HETATM	252	O	1	-9.625	-0.085	-6.287	O
HETATM	253	H	1	-9.375	-1.079	-6.109	H
HETATM	254	O	1	-8.354	3.292	-7.406	O
HETATM	255	O	1	-8.567	5.765	-6.803	O
HETATM	256	O	1	-9.272	4.132	-5.089	O
HETATM	257	O	1	-6.777	4.491	-5.781	O
HETATM	258	P	1	-8.217	4.411	-6.234	P
HETATM	259	H	1	-9.262	3.155	-4.705	H
HETATM	260	N	1	-1.046	3.182	-10.181	N
HETATM	261	H	1	-1.535	2.442	-10.029	H
HETATM	262	C	1	-1.703	4.413	-10.081	C
HETATM	263	N	1	-1.011	5.620	-10.121	N
HETATM	264	C	1	0.283	5.393	-10.381	C
HETATM	265	C	1	1.034	4.308	-10.521	C
HETATM	266	C	1	0.279	3.007	-10.491	C
HETATM	267	N	1	2.394	4.504	-10.731	N
HETATM	268	C	1	2.455	5.813	-10.781	C
HETATM	269	H	1	3.263	6.285	-10.996	H
HETATM	270	N	1	1.225	6.485	-10.491	N
HETATM	271	O	1	0.800	1.877	-10.521	O
HETATM	272	N	1	-3.001	4.417	-9.701	N
HETATM	273	H	1	-3.420	3.640	-9.520	H
HETATM	274	H	1	-3.447	5.195	-9.629	H
HETATM	275	C	1	1.017	8.013	-10.477	C
HETATM	276	H	1	0.553	8.319	-11.258	H
HETATM	277	C	1	0.229	8.407	-9.228	C
HETATM	278	H	1	-0.336	7.696	-8.917	H
HETATM	279	C	1	1.342	8.732	-8.238	C
HETATM	280	H	1	1.703	7.917	-7.882	H
HETATM	281	C	1	2.368	9.395	-9.125	C

HETATM	282	H	1	2.124	10.314	-9.264	H
HETATM	283	C	1	3.818	9.320	-8.630	C
HETATM	284	H	1	3.927	9.884	-7.862	H
HETATM	285	H	1	4.410	9.633	-9.318	H
HETATM	286	O	1	2.286	8.679	-10.385	O
HETATM	287	O	1	-0.510	9.574	-9.552	O
HETATM	288	H	1	-1.151	9.814	-8.756	H
HETATM	289	O	1	0.922	9.581	-7.170	O
HETATM	290	H	1	-0.093	9.437	-6.992	H
HETATM	291	O	1	4.147	7.965	-8.289	O
HETATM	292	O	1	6.629	7.917	-7.686	O
HETATM	293	O	1	5.079	8.789	-5.972	O
HETATM	294	O	1	5.174	6.271	-6.664	O
HETATM	295	P	1	5.246	7.711	-7.117	P
HETATM	296	H	1	4.106	8.882	-5.588	H
HETATM	297	N	1	3.274	0.708	-11.064	N
HETATM	298	H	1	2.590	1.271	-10.912	H
HETATM	299	C	1	4.567	1.232	-10.964	C
HETATM	300	N	1	5.695	0.418	-11.004	N
HETATM	301	C	1	5.334	-0.845	-11.264	C
HETATM	302	C	1	4.176	-1.479	-11.404	C
HETATM	303	C	1	2.961	-0.591	-11.374	C
HETATM	304	N	1	4.229	-2.852	-11.614	N
HETATM	305	C	1	5.525	-3.050	-11.664	C
HETATM	306	H	1	5.909	-3.902	-11.879	H
HETATM	307	N	1	6.321	-1.896	-11.374	N
HETATM	308	O	1	1.783	-0.992	-11.404	O
HETATM	309	N	1	4.706	2.523	-10.584	N
HETATM	310	H	1	3.978	3.020	-10.403	H
HETATM	311	H	1	5.527	2.885	-10.512	H
HETATM	312	C	1	7.863	-1.849	-11.360	C
HETATM	313	H	1	8.216	-1.420	-12.141	H
HETATM	314	C	1	8.337	-1.106	-10.111	C
HETATM	315	H	1	7.689	-0.471	-9.800	H
HETATM	316	C	1	8.544	-2.247	-9.121	C
HETATM	317	H	1	7.696	-2.521	-8.765	H
HETATM	318	C	1	9.096	-3.337	-10.008	C
HETATM	319	H	1	10.035	-3.191	-10.147	H
HETATM	320	C	1	8.870	-4.772	-9.513	C
HETATM	321	H	1	9.420	-4.939	-8.745	H
HETATM	322	H	1	9.119	-5.392	-10.201	H
HETATM	323	O	1	8.393	-3.180	-11.268	O
HETATM	324	O	1	9.575	-0.494	-10.435	O
HETATM	325	H	1	9.881	0.119	-9.639	H
HETATM	326	O	1	9.432	-1.918	-8.053	O
HETATM	327	H	1	9.395	-0.894	-7.875	H
HETATM	328	O	1	7.487	-4.957	-9.172	O
HETATM	329	O	1	7.181	-7.420	-8.569	O
HETATM	330	O	1	8.210	-5.970	-6.855	O
HETATM	331	O	1	5.696	-5.801	-7.547	O
HETATM	332	P	1	7.120	-6.023	-8.000	P
HETATM	333	H	1	8.404	-5.012	-6.471	H
HETATM	334	N	1	0.362	-3.330	-11.947	N
HETATM	335	H	1	0.994	-2.708	-11.795	H
HETATM	336	C	1	0.748	-4.670	-11.847	C
HETATM	337	N	1	-0.179	-5.708	-11.887	N
HETATM	338	C	1	-1.398	-5.216	-12.147	C

HETATM	339	C	1	-1.907	-3.999	-12.287	C
HETATM	340	C	1	-0.898	-2.883	-12.257	C
HETATM	341	N	1	-3.278	-3.907	-12.497	N
HETATM	342	C	1	-3.610	-5.176	-12.547	C
HETATM	343	H	1	-4.499	-5.469	-12.762	H
HETATM	344	N	1	-2.547	-6.089	-12.257	N
HETATM	345	O	1	-1.173	-1.669	-12.287	O
HETATM	346	N	1	2.018	-4.944	-11.467	N
HETATM	347	H	1	2.588	-4.272	-11.286	H
HETATM	348	H	1	2.292	-5.798	-11.395	H
HETATM	349	C	1	-2.661	-7.627	-12.243	C
HETATM	350	H	1	-2.271	-8.023	-13.024	H
HETATM	351	C	1	-1.972	-8.175	-10.994	C
HETATM	352	H	1	-1.272	-7.598	-10.683	H
HETATM	353	C	1	-3.128	-8.262	-10.004	C
HETATM	354	H	1	-3.311	-7.390	-9.648	H
HETATM	355	C	1	-4.269	-8.698	-10.891	C
HETATM	356	H	1	-4.222	-9.647	-11.030	H
HETATM	357	C	1	-5.673	-8.322	-10.396	C
HETATM	358	H	1	-5.896	-8.852	-9.628	H
HETATM	359	H	1	-6.316	-8.506	-11.084	H
HETATM	360	O	1	-4.040	-8.014	-12.151	O
HETATM	361	O	1	-1.492	-9.470	-11.318	O
HETATM	362	H	1	-0.915	-9.839	-10.522	H
HETATM	363	O	1	-2.893	-9.180	-8.936	O
HETATM	364	H	1	-1.871	-9.250	-8.758	H
HETATM	365	O	1	-5.712	-6.928	-10.055	O
HETATM	366	O	1	-8.130	-6.366	-9.452	O
HETATM	367	O	1	-6.795	-7.541	-7.738	O
HETATM	368	O	1	-6.365	-5.058	-8.430	O
HETATM	369	P	1	-6.735	-6.451	-8.883	P
HETATM	370	H	1	-5.863	-7.834	-7.354	H
HETATM	371	N	1	-3.350	-0.012	-12.830	N
HETATM	372	H	1	-2.797	-0.705	-12.678	H
HETATM	373	C	1	-4.723	-0.256	-12.730	C
HETATM	374	N	1	-5.658	0.775	-12.770	N
HETATM	375	C	1	-5.041	1.936	-13.030	C
HETATM	376	C	1	-3.777	2.314	-13.170	C
HETATM	377	C	1	-2.774	1.194	-13.140	C
HETATM	378	N	1	-3.543	3.669	-13.380	N
HETATM	379	C	1	-4.770	4.132	-13.430	C
HETATM	380	H	1	-4.969	5.046	-13.645	H
HETATM	381	N	1	-5.789	3.169	-13.140	N
HETATM	382	O	1	-1.537	1.341	-13.170	O
HETATM	383	N	1	-5.128	-1.490	-12.350	N
HETATM	384	H	1	-4.519	-2.127	-12.169	H
HETATM	385	H	1	-6.006	-1.673	-12.278	H
HETATM	386	C	1	-7.307	3.444	-13.126	C
HETATM	387	H	1	-7.741	3.097	-13.907	H
HETATM	388	C	1	-7.924	2.816	-11.877	C
HETATM	389	H	1	-7.423	2.059	-11.566	H
HETATM	390	C	1	-7.890	3.974	-10.887	C
HETATM	391	H	1	-7.003	4.066	-10.531	H
HETATM	392	C	1	-8.204	5.155	-11.774	C
HETATM	393	H	1	-9.153	5.207	-11.913	H
HETATM	394	C	1	-7.684	6.511	-11.279	C
HETATM	395	H	1	-8.187	6.789	-10.511	H

HETATM	396	H	1	-7.799	7.171	-11.967	H
HETATM	397	O	1	-7.548	4.856	-13.034	O
HETATM	398	O	1	-9.263	2.474	-12.201	O
HETATM	399	H	1	-9.690	1.938	-11.405	H
HETATM	400	O	1	-8.827	3.837	-9.819	O
HETATM	401	H	1	-9.003	2.828	-9.641	H
HETATM	402	O	1	-6.293	6.405	-10.938	O
HETATM	403	O	1	-5.481	8.751	-10.335	O
HETATM	404	O	1	-6.790	7.546	-8.621	O
HETATM	405	O	1	-4.365	6.859	-9.313	O
HETATM	406	P	1	-5.712	7.372	-9.766	P
HETATM	407	H	1	-7.178	6.649	-8.237	H
HETATM	408	N	1	0.339	3.333	-13.713	N
HETATM	409	H	1	-0.409	2.856	-13.561	H
HETATM	410	C	1	0.239	4.724	-13.613	C
HETATM	411	N	1	1.362	5.545	-13.653	N
HETATM	412	C	1	2.452	4.811	-13.913	C
HETATM	413	C	1	2.697	3.515	-14.053	C
HETATM	414	C	1	1.478	2.634	-14.023	C
HETATM	415	N	1	4.019	3.140	-14.263	N
HETATM	416	C	1	4.608	4.312	-14.313	C
HETATM	417	H	1	5.537	4.414	-14.528	H
HETATM	418	N	1	3.757	5.426	-14.023	N
HETATM	419	O	1	1.494	1.389	-14.053	O
HETATM	420	N	1	-0.945	5.256	-13.233	N
HETATM	421	H	1	-1.643	4.716	-13.052	H
HETATM	422	H	1	-1.036	6.148	-13.161	H
HETATM	423	C	1	4.189	6.907	-14.009	C
HETATM	424	H	1	3.889	7.375	-14.790	H
HETATM	425	C	1	3.628	7.587	-12.760	C
HETATM	426	H	1	2.824	7.167	-12.449	H
HETATM	427	C	1	4.777	7.431	-11.770	C
HETATM	428	H	1	4.775	6.540	-11.414	H
HETATM	429	C	1	5.984	7.620	-12.657	C
HETATM	430	H	1	6.135	8.558	-12.796	H
HETATM	431	C	1	7.279	6.961	-12.162	C
HETATM	432	H	1	7.608	7.433	-11.394	H
HETATM	433	H	1	7.947	7.007	-12.850	H
HETATM	434	O	1	5.618	6.999	-13.917	O
HETATM	435	O	1	3.428	8.953	-13.084	O
HETATM	436	H	1	2.941	9.434	-12.288	H
HETATM	437	O	1	4.739	8.378	-10.702	O
HETATM	438	H	1	3.753	8.659	-10.524	H
HETATM	439	O	1	7.028	5.589	-11.821	O
HETATM	440	O	1	9.276	4.536	-11.218	O
HETATM	441	O	1	8.215	5.964	-9.504	O
HETATM	442	O	1	7.278	3.624	-10.196	O
HETATM	443	P	1	7.929	4.910	-10.649	P
HETATM	444	H	1	7.363	6.444	-9.120	H
HETATM	445	N	1	3.279	-0.685	-14.596	N
HETATM	446	H	1	2.883	0.108	-14.444	H
HETATM	447	C	1	4.673	-0.732	-14.496	C
HETATM	448	N	1	5.373	-1.934	-14.536	N
HETATM	449	C	1	4.529	-2.942	-14.796	C
HETATM	450	C	1	3.214	-3.049	-14.936	C
HETATM	451	C	1	2.465	-1.745	-14.906	C
HETATM	452	N	1	2.703	-4.325	-15.146	N

HETATM	453	C	1	3.807	-5.033	-15.196	C
HETATM	454	H	1	3.811	-5.968	-15.411	H
HETATM	455	N	1	5.004	-4.303	-14.906	N
HETATM	456	O	1	1.225	-1.631	-14.936	O
HETATM	457	N	1	5.326	0.391	-14.116	N
HETATM	458	H	1	4.862	1.141	-13.935	H
HETATM	459	H	1	6.223	0.388	-14.044	H
HETATM	460	C	1	6.431	-4.888	-14.892	C
HETATM	461	H	1	6.928	-4.639	-15.673	H
HETATM	462	C	1	7.166	-4.402	-13.643	C
HETATM	463	H	1	6.833	-3.558	-13.332	H
HETATM	464	C	1	6.891	-5.528	-12.653	C
HETATM	465	H	1	6.005	-5.433	-12.297	H
HETATM	466	C	1	6.953	-6.748	-13.540	C
HETATM	467	H	1	7.870	-6.996	-13.679	H
HETATM	468	C	1	6.162	-7.967	-13.045	C
HETATM	469	H	1	6.597	-8.343	-12.277	H
HETATM	470	H	1	6.138	-8.635	-13.733	H
HETATM	471	O	1	6.373	-6.319	-14.800	O
HETATM	472	O	1	8.546	-4.345	-13.967	O
HETATM	473	H	1	9.075	-3.911	-13.171	H
HETATM	474	O	1	7.836	-5.589	-11.585	O
HETATM	475	H	1	8.219	-4.638	-11.407	H
HETATM	476	O	1	4.824	-7.574	-12.704	O
HETATM	477	O	1	3.542	-9.699	-12.101	O
HETATM	478	O	1	5.072	-8.793	-10.387	O
HETATM	479	O	1	2.844	-7.617	-11.079	O
HETATM	480	P	1	4.054	-8.399	-11.532	P
HETATM	481	H	1	5.639	-7.997	-10.003	H
HETATM	482	N	1	-1.024	-3.190	-15.479	N
HETATM	483	H	1	-0.194	-2.878	-15.327	H
HETATM	484	C	1	-1.216	-4.571	-15.379	C
HETATM	485	N	1	-2.485	-5.141	-15.419	N
HETATM	486	C	1	-3.399	-4.196	-15.679	C
HETATM	487	C	1	-3.368	-2.877	-15.819	C
HETATM	488	C	1	-1.993	-2.269	-15.789	C
HETATM	489	N	1	-4.584	-2.236	-16.029	N
HETATM	490	C	1	-5.403	-3.260	-16.079	C
HETATM	491	H	1	-6.334	-3.166	-16.294	H
HETATM	492	N	1	-4.803	-4.526	-15.789	N
HETATM	493	O	1	-1.750	-1.048	-15.819	O
HETATM	494	N	1	-0.168	-5.338	-14.999	N
HETATM	495	H	1	0.627	-4.955	-14.818	H
HETATM	496	H	1	-0.265	-6.229	-14.927	H
HETATM	497	C	1	-5.533	-5.885	-15.775	C
HETATM	498	H	1	-5.338	-6.405	-16.556	H
HETATM	499	C	1	-5.127	-6.667	-14.526	C
HETATM	500	H	1	-4.252	-6.424	-14.215	H
HETATM	501	C	1	-6.218	-6.276	-13.536	C
HETATM	502	H	1	-6.031	-5.404	-13.180	H
HETATM	503	C	1	-7.438	-6.209	-14.423	C
HETATM	504	H	1	-7.781	-7.095	-14.562	H
HETATM	505	C	1	-8.567	-5.296	-13.928	C
HETATM	506	H	1	-8.987	-5.689	-13.160	H
HETATM	507	H	1	-9.230	-5.201	-14.616	H
HETATM	508	O	1	-6.951	-5.678	-15.683	O
HETATM	509	O	1	-5.215	-8.045	-14.850	O

HETATM	510	H	1	-4.838	-8.616	-14.054	H
HETATM	511	O	1	-6.377	-7.209	-12.468	O
HETATM	512	H	1	-5.471	-7.689	-12.290	H
HETATM	513	O	1	-8.036	-4.006	-13.587	O
HETATM	514	O	1	-10.017	-2.509	-12.984	O
HETATM	515	O	1	-9.275	-4.125	-11.270	O
HETATM	516	O	1	-7.872	-2.032	-11.962	O
HETATM	517	P	1	-8.776	-3.154	-12.415	P
HETATM	518	H	1	-8.542	-4.772	-10.886	H
HETATM	519	N	1	-3.065	1.352	-16.362	N
HETATM	520	H	1	-2.842	0.494	-16.210	H
HETATM	521	C	1	-4.419	1.687	-16.262	C
HETATM	522	N	1	-4.853	3.009	-16.302	N
HETATM	523	C	1	-3.818	3.819	-16.562	C
HETATM	524	C	1	-2.509	3.651	-16.702	C
HETATM	525	C	1	-2.048	2.219	-16.672	C
HETATM	526	N	1	-1.745	4.793	-16.912	N
HETATM	527	C	1	-2.677	5.714	-16.962	C
HETATM	528	H	1	-2.487	6.630	-17.177	H
HETATM	529	N	1	-4.000	5.250	-16.672	N
HETATM	530	O	1	-0.859	1.850	-16.702	O
HETATM	531	N	1	-5.291	0.725	-15.882	N
HETATM	532	H	1	-4.993	-0.106	-15.701	H
HETATM	533	H	1	-6.167	0.914	-15.810	H
HETATM	534	C	1	-5.274	6.118	-16.658	C
HETATM	535	H	1	-5.812	5.978	-17.439	H
HETATM	536	C	1	-6.094	5.795	-15.409	C
HETATM	537	H	1	-5.944	4.900	-15.098	H
HETATM	538	C	1	-5.591	6.840	-14.419	C
HETATM	539	H	1	-4.744	6.563	-14.063	H
HETATM	540	C	1	-5.398	8.046	-15.306	C
HETATM	541	H	1	-6.243	8.480	-15.445	H
HETATM	542	C	1	-4.371	9.074	-14.811	C
HETATM	543	H	1	-4.718	9.532	-14.043	H
HETATM	544	H	1	-4.208	9.723	-15.499	H
HETATM	545	O	1	-4.920	7.506	-16.566	O
HETATM	546	O	1	-7.456	6.027	-15.733	O
HETATM	547	H	1	-8.064	5.712	-14.937	H
HETATM	548	O	1	-6.503	7.096	-13.351	O
HETATM	549	H	1	-7.075	6.245	-13.173	H
HETATM	550	O	1	-3.144	8.411	-14.470	O
HETATM	551	O	1	-1.448	10.224	-13.867	O
HETATM	552	O	1	-3.133	9.655	-12.153	O
HETATM	553	O	1	-1.198	8.041	-12.845	O
HETATM	554	P	1	-2.220	9.058	-13.298	P
HETATM	555	H	1	-3.853	8.994	-11.769	H
